# Lessons from a pre-specified meta-analysis of sodium-glucose cotransporter-2 (SGLT2) inhibitors in heart failure: Time for new clinical recommendations

**DOI:** 10.21542/gcsp.2023.14

**Published:** 2023-05-11

**Authors:** Susy Kotit

**Affiliations:** 1 Aswan Heart Centre (AHC), Aswan, Egypt

## Abstract

Introduction: Cardiovascular disease remains the leading cause of death worldwide with heart failure (HF) being one of the significant contributors to morbidity and mortality. The incidence of HF with preserved ejection fraction (HFpEF) is increasing, especially in young adults making it a growing public health matter. Sodium–glucose cotransporter-2 (SGLT2) inhibitors have been shown to reduce the development, progression, and mortality of heart failure in patients with reduced EF regardless of patients’ diabetes status but their clinical benefits in patients with heart failure and preserved ejection fraction are less well-established. Recent trials have shown reductions in cardiovascular death and heart failure events in patients with mildly reduced or preserved ejection fraction (EF), although with uncertainty around the consistency of clinical benefits across the classes and therapeutic effects.

Study and Results: The meta-analysis used data from trials on patients with mildly reduced or preserved EF (DELIVER and EMPEROR-Preserved), reduced EF (DAPA-HF and EMPEROR-Reduced), and those hospitalized (SOLOIST-WHF). The endpoints evaluated included a composite of time to cardiovascular (CV) death or first hospitalization for heart failure, cardiovascular death, all-cause death, first and recurrent heart failure hospitalizations, and urgent heart failure visits (not requiring hospitalization). Among 12251 participants in the DELIVER and EMPEROR-Preserved trials, SGLT2 inhibitors reduced composite cardiovascular death or first hospitalization for HF (HR 0.80 [95% CI 0.73–0.87]) with consistent reductions in both components: cardiovascular death (HR 0.88 [95% CI 0.77–1.00]) and first hospitalization for HF (HR 0.74 [95% CI 0.67–0.83]). In the broader analysis of the five trials with a total of 21 947 participants, SGLT2 inhibitors reduced the risk of composite cardiovascular death or hospitalization for HF (HR 0.77 [95% CI 0.72–0.82]), cardiovascular death (0.87 [0.79–0.95]), first hospitalization for heart failure (HR 0.72 [95% CI 0.67–0.78]), and all-cause mortality (HR 0.92 [95% CI 0.86–0.99]). These treatment effects for each of the studied endpoints were consistently observed across all five trials and across the HF subgroups, including those on mildly reduced or preserved ejection fraction.

Lessons learned: SGLT2 inhibitors significantly reduce the risk of mortality and worsening of heart failure and improve patient symptoms and overall health status across the full spectrum of ejection fraction. SGLT2 inhibitors should be considered foundational therapy in all patients with heart failure, irrespective of LVEF or care setting. The results presented propose an update of the recommendations for the pharmacological treatment of heart failure, to prioritize the use of SGLT2 inhibitors in patients across the full EF spectrum. Future investigations should include the long-term benefits of the use of SGLT2 inhibitors among the different HF subgroups, including the performance of SGLT2 inhibitors in those excluded from the current heart failure trials.

## Introduction

Cardiovascular disease remains the leading cause of death worldwide. Heart failure (HF) is one of the most significant contributors to morbidity and mortality, with a lifetime risk ranging from 20% to 45% after 45 years of age, varying across racial and ethnic groups^1^. Trends show that the incidence of HF is increasing due to the escalation in the prevalence of hypertension, obesity, atrial fibrillation, diabetes, and the growing elderly segment of the general population^2^. In addition, HF with preserved ejection fraction (HFpEF; EF > 50%) is becoming more prevalent^1^, with a significant increase observed in young adults in recent years^3–5^, possibly related to the rising burden of cardiometabolic risk factors beginning in young adulthood^6^ but also as a sequela of COVID-19^7–11^. Heart failure diminishes the quality of life and increases hospitalization, leading to a potential economic burden stemming from the loss of productivity years and healthcare utilization associated with HF morbidity and mortality at a young age^5,12^, making it a growing public health matter^13^.

**Figure 1.**
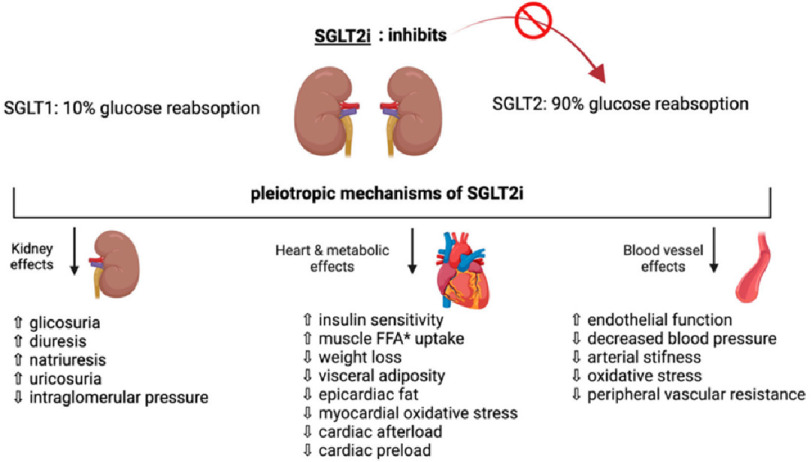
Pleiotropic effects of SGLT2i: Sodium–glucose co-transporter 2 (SGLT2) inhibitors were originally developed as anti-hyperglycemic drugs. However, independently of their actions on blood glucose, these drugs exert a broad range of biological effects including actions to inhibit cardiac inflammation and fibrosis, as well as to antagonize sodium retention and improve glomerular function, affecting the principal pathophysiological derangements in HFpEF. ^30–34^ SGLT2 inhibitors exert favorable effects in experimental models of HFpEF ^35^ and recent evidence supports the efficacy of SGLT2i in reducing cardiovascular complications and hospitalization in patients with and without diabetes by ameliorating renal, cardiometabolic, and vascular effects. (*FFA: free fatty acid) ^36–43^.

Sodium–glucose cotransporter-2 (SGLT2) inhibitors (Figure 1) have been shown to reduce the development and progression of heart failure in patients with reduced ejection fraction (HFrEF; EF ≤ 40%), leading to a decrease in the number of cardiovascular deaths and hospitalizations, regardless of patients’ diabetes status (see e.g., DAPA-HF^14,15^ and EMPEROR-REDUCED^16–19^) even in previously hospitalized patients (SOLOIST-WHF)^20^. Currently, SGLT2 inhibitors are established as standard care in the treatment of patients with HFrEF^21,22^, but their clinical benefits in patients with heart failure and preserved ejection fraction are less well established.

Recently, the DELIVER^23^ and EMPEROR-Preserved^24,25^ trials showed reductions in composite cardiovascular death and heart failure events in patients with mildly reduced or preserved ejection fraction, supporting the use of the SGLT2 inhibitors in this patient population^26,27^. SGLT2 inhibitors therefore represent a possible important advance in the treatment of HFpEF, either alone or in combination with mineralocorticoid receptor antagonists (MRAs) and angiotensin receptor neprilysin inhibitors (ARNIs). However, whether the clinical benefits of SGLT2 inhibitors in heart failure extend to all subpopulations, including those at the highest end of the ejection fraction spectrum^28^ and those already treated with other therapies commonly used in heart failure^29^, has not been clarified.

Thus, recommendations for SGLT2 inhibitors in heart failure with mildly reduced and preserved ejection fraction remain absent, partly due to uncertainty around the consistency of clinical benefits across the HF classes and therapeutic effects, particularly cardiovascular death.

### A pre-specified meta-analysis of patients with chronic HF from 5 randomized clinical trials: DELIVER, EMPEROR-Preserved, DAPA-HF, EMPEROR-Reduced, and SOLOIST-WHF

**Figure 2.**
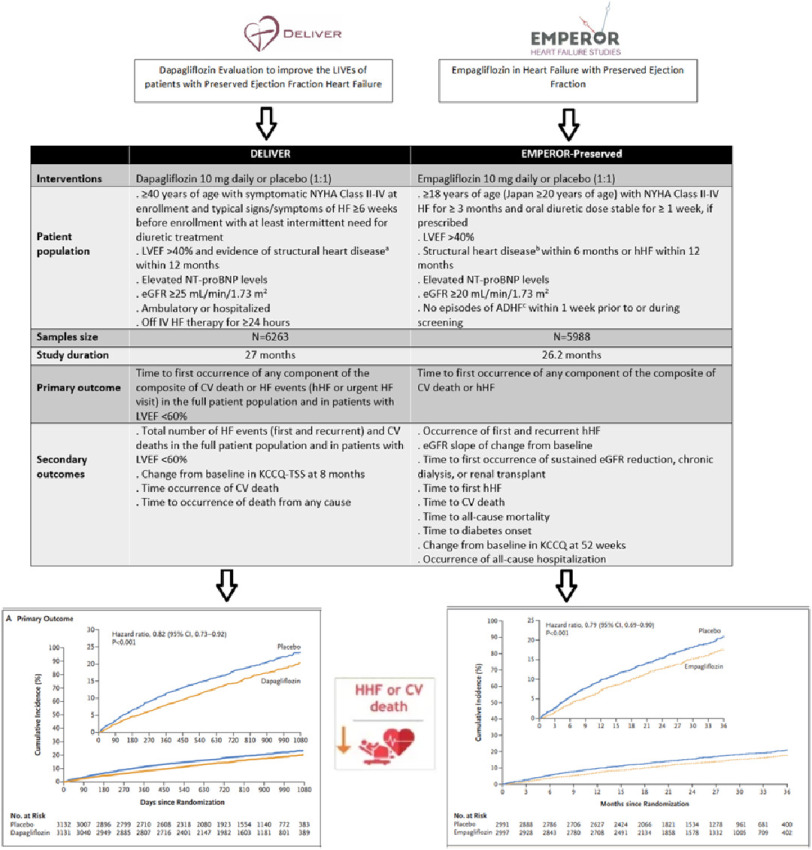
Study design of DELIVER and EMPEROR-Preserved trials and primary endpoint (heart failure hospitalization or CV death) results. ^a^Structural heart disease was defined as: 1) LA enlargement with at least one of the following: LA width (diameter) ≥ 3.8 cm or LA length ≥ 5.0 cm, or LA area ≥ 20 cm, or LA volume ≥ 55 mL or LA volume index ≥ 29 mL/m.; 2) Left ventricular hypertrophy with septal thickness or posterior wall thickness ≥ 1.1 cm. ^44^ ^23^; ^b^evidence of structural changes in the heart (as evidenced by increases in left atrial size or left ventricular mass) on echocardiography ^24^; ^c^(ADHF) Acute decompensated heart failure; (KCCQ) Kansas City Cardiomyopathy Questionnaire; (hHF) hospitalization for HF.

**Figure 3.**
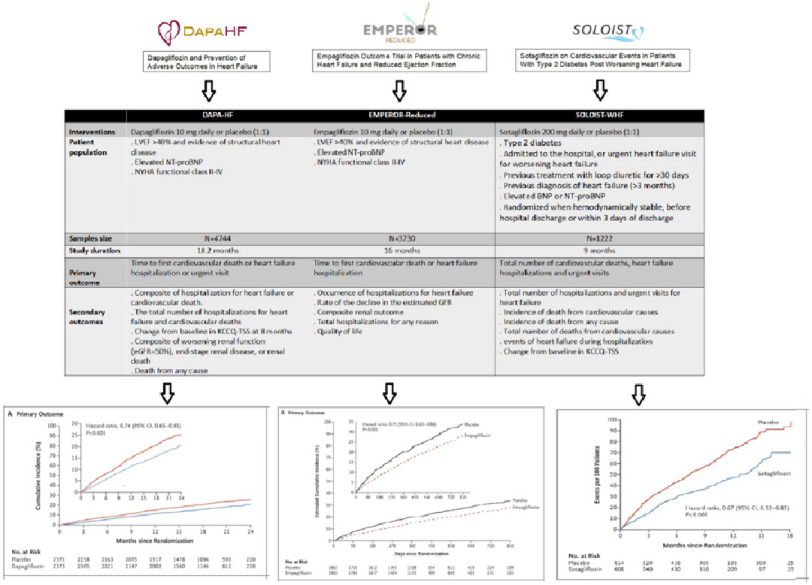
Study design of DAPA-HF, EMPEROR-Reduced and SOLOIST-WHF trials and primary endpoint (heart failure hospitalization or CV death) results.

The prespecified meta-analysis of the two largest trials of heart failure with mildly reduced or preserved ejection fraction used participant-level data from DELIVER^23^ and trial-level data from EMPEROR-Preserved^24^ and employed harmonized definitions of endpoints and subgroups (Figure 2). The meta-analysis was extended to include trials in patients with reduced ejection fractions (DAPA-HF)^14^ and EMPEROR-Reduced^16^) and those admitted to hospital with worsening heart failure, enrolled with any ejection fraction (SOLOIST-WHF) (Figure 3)^20^.

**Figure 4.**
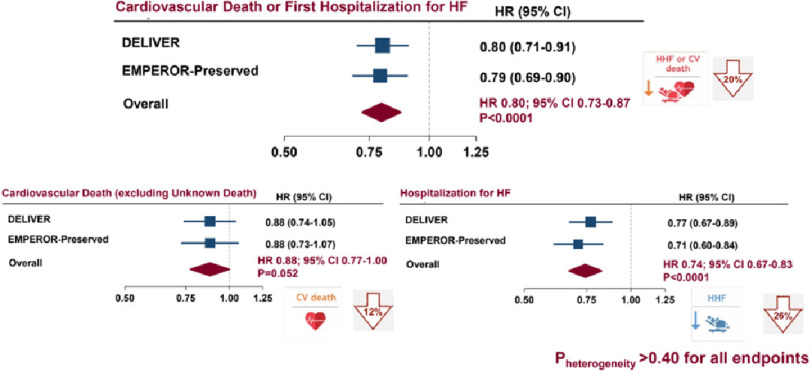
DELIVER and EMPEROR-Preserved meta-analysis^45,46^.

**Figure 5.**
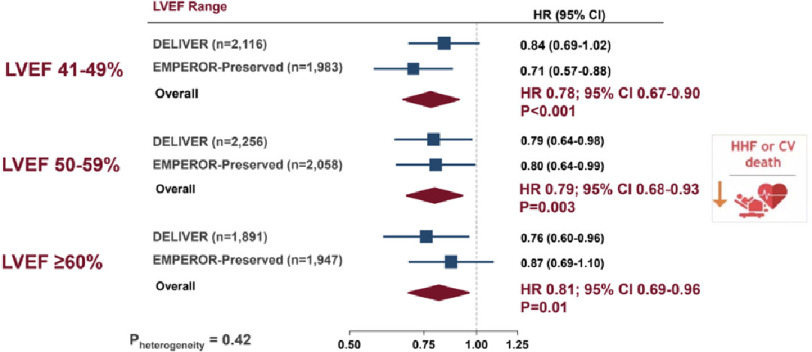
DELIVER and EMPEROR-Preserved meta-analysis: consistent reductions in primary endpoint (heart failure hospitalization or CV death) across LVEF range, including LVEF ≥60%^45,46^.

 The study aimed at clarifying the effect of SGLT2 inhibitor use in patients with heart failure (HF) on HF hospitalizations, mortality, and health status across different subgroups as seen in five randomized controlled trials.

The primary endpoint of the meta-analysis was a composite of time to cardiovascular (CV) death or first hospitalization for heart failure. The secondary endpoints evaluated included cardiovascular death, all-cause death, first and recurrent heart failure hospitalizations and urgent heart failure visits (not requiring hospitalization).

Changes in health status and quality of life from baseline to 8 months were determined using the Kansas City Cardiomyopathy Questionnaire (KCCQ). The treatment effects of SGLT2 inhibitors were assessed across 14 subgroups, which included LVEF, history of diabetes, age, sex, race, geographical region, KCCQ total symptom score, body mass index, estimated glomerular filtration rate (eGFR), history of atrial fibrillation or flutter, New York Heart Association (NYHA) functional class, hospitalization for HF within 12 months, N-terminal pro–B-type natriuretic peptide (NT-proBNP) concentration, baseline use of mineralocorticoid receptor antagonists (MRAs), and baseline use of angiotensin receptor neprilysin inhibitors (ARNIs).

## Results

The meta-analysis was performed in two stages. Stage 1 consisted of a meta-analysis of the DELIVER and EMPEROR-Preserved trials on patients with mildly reduced or preserved EF. In stage 2 the analysis was extended to include patients with HFrEF and in the hospitalized setting (DAPA-HF, EMPEROR-Reduced and SOLOIST-WHF).

### DELIVER and EMPEROR-Preserved

Among 12,251 patients from the DELIVER and EMPEROR-Preserved trials, there was a significant reduction in the primary endpoint for composite CV death or first hospitalization for HF for patients receiving an SGLT2 inhibitor compared to placebo (HR, 0.80; 95% CI [0.73–0.87]) (Figure 4).

Results were consistent for cardiovascular death (HR, 0.88; 95% CI [0.77–1.00]), first HF hospitalization (HR, 0.74; 95% CI [0.67–0.83]) and worsening heart failure events (HF hospitalizations and urgent visits) (HR, 0.80, 95% CI [0.73–0.87]). No effect on death from any cause was found (HR, 0.97; 95% CI [0.88–1.06]). Adverse events were infrequent and well-balanced between groups, although less frequent in the SGLT2 inhibitor groups (Figure 5).

### DELIVER, EMPEROR-Preserved, DAPA-HF, EMPEROR-Reduced and SOLOIST-WHF

A total of 21,947 participants were analyzed across the five trials. Median follow-up time ranged from 9 months to 2.3 years. Patients in trials of HFrEF were younger and more frequently males. Most patients were in NYHA functional class II.

Baseline median NT-proBNP across the trials ranged from 974 pg/mL to 1910 pg/mL. Median eGFR was lowest in SOLOIST-WHF (50 mL/min/1.73 m^2^). There were differences in background medical treatment according to ejection fraction, with greater use of ARNIs and MRAs in patients with reduced ejection fraction.

The rates of incident hospitalization for heart failure, cardiovascular death, and all-cause mortality were higher in trials enrolling outpatients with heart failure with reduced ejection fraction than in those enrolling patients with heart failure with mildly reduced or preserved ejection fraction, and the highest event rates were reported in the SOLOIST-WHF trial, as patients were randomly assigned following an episode of worsening heart failure.

Treatment with an SGLT2 inhibitor was shown to reduce the risk of cardiovascular death or hospitalization for heart failure (HR, 0.77; 95% CI [0.72–0.82]), with an NNT of 25 (20–31) over a weighted mean of 23 months’ follow-up. Reductions were also seen in the key secondary endpoints of CV death (HR, 0.87; 95% CI [0.79–0.95]); NNT 88 [54–229]), first hospitalization for heart failure (HR, 0.72; 95% CI [0.67–0.78]); NNT of 28 (24–35)), and all-cause death (HR, 0.92; 95% CI [0.86–0.99]); NNT 92 [52–733]).

SGLT2 inhibitor use was associated with more participants achieving clinically meaningful improvements and fewer having clinically meaningful deterioration in KCCQ scores by 8 months.

The effect of SGLT2 inhibitors on the composite of cardiovascular death or first hospitalization for heart failure was consistent across 14 clinically relevant subgroups, except for NYHA functional classification, (attenuated effect with NYHA III or IV compared to NYHA class II patients [HR, 0.86; 95% CI [0.77–0.95]]). However, the effect of SGLT2 inhibitor treatment was similar across baseline KCCQ total symptom score (*p*-value for heterogeneity = 0.98).

**Figure 6.**
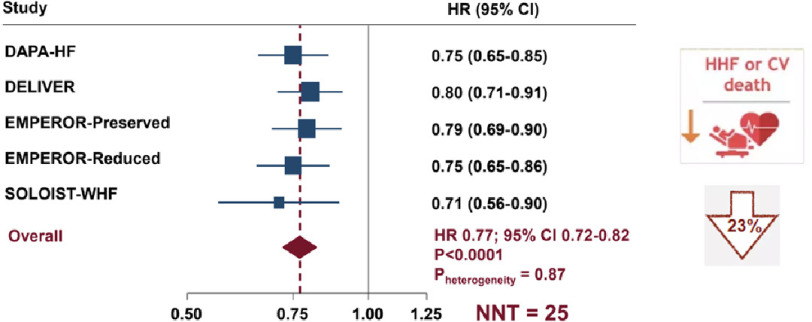
Meta-analysis of 5 large placebo-controlled trials: relative risk reduction of primary endpoint (CV death or HF hospitalization) ^45,46^.

Consistent benefits were seen across ejection fraction groups: EF ≤ 40% (HR 0.75 [95% CI 0.68–0.83]), EF = 41–49% (HR 0.78 [95% CI 0.67–0.90]), EF = 50–59% (HR 0.79 [95% CI 0.68–0.93]), and EF = 60% (HR 0.81 [95% CI 0.69–0.96]) (Figure 6).

## Discussion

The meta-analysis of the DELIVER and EMPEROR-Preserved trials showed that the SGLT2 inhibitors dapagliflozin and empagliflozin similarly and robustly reduced cardiovascular death or hospitalization for heart failure among patients with mildly reduced and preserved ejection fraction compared with placebo.

The more extensive examination of data on the use SGLT2 inhibitors in over 20,000 participants in five trials, shows reduced risk of HF hospitalization and CV and all-cause mortality across a broad range of patients with heart failure, irrespective of LVEF, care setting or concomitant treatment with an MRA or ARNI.

The greatest benefit of the addition of an SGLT2 inhibitor to standard therapy in patients with heart failure was a 28% relative reduction in the risk of hospitalization for heart failure, with an NNT of 28 to prevent one event over a follow-up of 23 months. Although smaller, the effect on mortality was significant. These estimates for reductions in cardiovascular death are highly concordant with those observed in other patient populations, such as those with type 2 diabetes^47^.

Furthermore, patients treated with SGLT2 inhibitors were 10–20% more likely to have improvements in health status and, conversely were 10–20% less likely to face important deterioration in health status compared with patients in control groups.

In addition, there were benefits of SGLT2 inhibitors on meaningful clinical events, symptom burden, and overall health status in patients with heart failure as SGLT2 inhibitors ameliorate symptoms and confer clinically meaningful improvements in health-related quality of life as seen in previous trials^48,49^.

The new evidence on the benefits of SGLT2 inhibitors in heart failure with mildly reduced or preserved ejection fraction, along with their favorable safety profile, the minimal requirement for monitoring, rapid onset of benefit, and beneficial effects on kidney function, supports prioritizing initiation of SGLT2 inhibitors in all HF patients^26,50^. The results presented should promote an update of the recommendations for pharmacological treatment of heart failure in mildly reduced and preserved EF to include the use of SGLT2 inhibitors in patients with HF across the full spectrum of ejection fraction, irrespective of diabetes status and care setting and regardless of background therapies^51^.

## Limitations

Although the meta-analysis of DELIVER and EMPEROR-Preserved was prespecified and preregistered, the supportive five-trial meta-analysis was done post hoc, which may mask real clinical benefit. The provided results should therefore be treated with skepticism irrespective of their statistical significance.

The individual participant level data from the EMPEROR trials and SOLOIST-WHF were not accessed and the analysis relies on published data only which might have affected the quality and integrity of the data analyzed. Furthermore, subgroup data for the outcomes of interest were not available for the SOLOIST-WHF trial.

It is uncertain if the results are generalizable, due to racial underrepresentation of some population groups and the exclusion of patients with severe kidney disfunction. Urgent heart failure visits were not centrally adjudicated in the EMPEROR-Preserved trial. Although definitions of most other efficacy endpoints were aligned, safety event definitions could not be reconciled because of differential timeframes of assessment and data ascertainment.

There was no statistical heterogeneity across the five trials for any endpoint and thus the clinical benefits of the tested therapies are assumed to be similar. However, the possibility that select differences in clinical efficacy and safety might still exist cannot be excluded.

## Lessons learned

SGLT2 inhibitors significantly reduce the risk of mortality and worsening of heart failure and improve patient symptoms and overall health status across the full spectrum of ejection fraction when added to standard heart failure therapy. SGLT2 inhibitors should be considered foundational therapy in all patients with heart failure, irrespective of LVEF or care setting in order to help prevent hospitalization, morbidity and mortality and to extend meaningful survival and improve health-related quality of life.

The results presented propose an update of the recommendations of pharmacological treatment of heart failure, to prioritize the use of SGLT2 inhibitors in patients with HF across the full EF spectrum.

The long-term benefits of the use of SGLT2 inhibitors should be studied in detail among the different HF subgroups and future investigations should include performance of SGLT2 inhibitors in those excluded from the current heart failure trials such as patients with amyloid cardiomyopathy, genetic hypertrophic or obstructive cardiomyopathy, primary uncorrected valvular disease or severe kidney disease.
